# Correlation of Circulating CD133+ Progenitor Subclasses with a Mild Phenotype in Duchenne Muscular Dystrophy Patients

**DOI:** 10.1371/journal.pone.0002218

**Published:** 2008-05-21

**Authors:** Chiara Marchesi, Marzia Belicchi, Mirella Meregalli, Andrea Farini, Alessandra Cattaneo, Daniele Parolini, Manuela Gavina, Laura Porretti, Maria Grazia D'Angelo, Nereo Bresolin, Giulio Cossu, Yvan Torrente

**Affiliations:** 1 Stem cell Laboratory, Department of Neurological Science, Fondazione Istituto di Ricovero e Cura a Carattere Scientifico (IRCCS) Ospedale Maggiore Policlinico, Centro Dino Ferrari, University of Milan, Milan, Italy; 2 Centro Trasfusionale e di Immunologia dei Trapianti, Fondazione Istituto di Ricovero e Cura a Carattere Scientifico (IRCCS) Ospedale Maggiore Policlinico, University of Milan, Milan, Italy; 3 Istituto di Ricovero e Cura a Carattere Scientifico (IRCCS) E. Medea, La Nostra Famiglia, Bosisio Parini, Italy; 4 Stem Cell Research Institute, San Raffaele Hospital, Milan, Italy; Fred Hutchinson Cancer Research Center, United States of America

## Abstract

**Background:**

Various prognostic serum and cellular markers have been identified for many diseases, such as cardiovascular diseases and tumor pathologies. Here we assessed whether the levels of certain stem cells may predict the progression of Duchenne muscular dystrophy (DMD).

**Methods and Findings:**

The levels of several subpopulations of circulating stem cells expressing the CD133 antigen were determined by flow cytometry in 70 DMD patients. The correlation between the levels and clinical status was assessed by statistical analysis. The median (±SD) age of the population was 10.66±3.81 (range 3 to 20 years). The levels of CD133+CXCR4+CD34- stem cells were significantly higher in DMD patients compared to healthy controls (mean±standard deviation: 17.38±1.38 vs. 11.0±1.70; P = 0.03) with a tendency towards decreased levels in older patients. Moreover, the levels of this subpopulation of cells correlated with the clinical condition. In a subgroup of 19 DMD patients after 24 months of follow-up, increased levels of CD133+CXCR4+CD34- cells was shown to be associated with a phenotype characterised by slower disease progression. The circulating CD133+CXCR4+CD34- cells in patients from different ages did not exhibit significant differences in their myogenic and endothelial in vitro differentiation capacity.

**Conclusions:**

Our results suggest that levels of CD133+CXCR4+CD34- could function as a new prognostic clinical marker for the progression of DMD.

## Introduction

The progression of Duchenne muscular dystrophy (DMD) is characterized by a progressive impairment of muscle function leading to death due to cardio-pulmonary failure in the late teens or early twenties [1], [2], [3]. DMD exhibits a spectrum of muscle disease, and clinical variability is observed regarding age of onset, patterns of skeletal muscle involvement, heart damage, and rate of progression. Unfortunately, most therapeutic strategies for DMD have been palliative rather than curative. The reading frame hypothesis has been proposed to explain the phenotypic differences between Duchenne and Becker patients, as the DMD phenotype results from deletions that shift the translational reading frame while the BMD phenotype results from deletions that maintain the translational reading frame. However, several exceptions have been previously noted in the literature. Moreover, among DMD patients, the clinical course of the disease and response to steroid treatment cannot be entirely predicted based on the type of mutation. This indicates that genotype alone cannot predict disease progression, and that other genetic or epigenetic factors determine the clinical expression of this disorder [4]–[5]. The development of reliable prognostic markers for the progression of the disease or response to treatment would not only be advantageous for patient treatment, but also help facilitate the evaluation of new experimental approaches in DMD.

To date, several studies have attempted to identify prognostic factors to predict the progression of DMD, and in this context, patients have been clinically assessed based on cardiac and pulmonary function measurements and their rate of decline [6]–[8]. However, a precise assessment of clinical severity and prediction of progression remains challenging. The levels of circulating endothelial stem cells are considered a strong biomarker of cardiovascular risk and have been recently used as a surrogate end point in primary intervention studies of risk reduction [9]–[14]. In addition, obstructive and restrictive lung diseases seem to be associated with variations in the number of endothelial progenitor cells in peripheral blood [15]. These studies are based on the growing evidence that circulating endothelial progenitor cells have the capacity to repair damaged endothelium and generate new blood vessels in the tissue damaged area. Recently, we identified a subpopulation of human circulating stem cells expressing the CD133 antigen that can differentiate into endothelial and muscle cell types [16]. These data confirmed and extended the previous reports by Miraglia and Gallacher, who characterized circulating CD133+ cells as having hematopoietic and endothelial potential [17]–[18]. To test whether the levels of circulating stem cells expressing the CD133 antigen could predict the clinical severity and progression of DMD, we assessed stem cell levels in DMD patients and analyzed the potential correlation with clinical results.

## Materials and Methods

### Isolation and characterization of human CD133+ subpopulations from normal healthy and dystrophic blood tissues

This study was approved by the “Ethical Committee of the IRCCS Eugenio Medea Bosisio Parini”. Blood was obtained from 30 healthy volunteers (3–20 years of age) and 70 dystrophic patients (3–25 years of age) after written informed consent was obtained from each patient. Samples were obtained from routine blood tests performed in healthy and DMD subjects. Blood samples were diluted 1∶3 in Iscove's modified Dulbecco's medium (IMDM) (Gibco Life Technologies, Grand Island, NY). The mononuclear cells were collected by centrifugation (Ficoll-Hypaque, Pharmacia Biotech, Uppsala, Sweden) and incubated with CD133-conjugated super paramagnetic microbeads (monoclonal antibody, MoAb; CD133 Isolation Kit, Miltenyi Biotec, Bergisch-Gladbach, Germany). Beads were washed and processed through a MACS magnetic separation column (Miltenyi Biotec) to obtain purified CD133^+^ cells. After selection, an aliquot of the CD133^+^ cell fraction was analyzed to assess purity, which was determined for each isolation experiment. For four-color flow cytometric analysis, 5×10^4^ cells were incubated with anti-CD133-phycoerythrin (PE) (Miltenyi Biotec), anti-CD34APC (Pharmingen), anti-CXCR4-PECY5 (Pharmingen), anti-CDw90 (Thy-1)-fluorescein-isotiocyanate (FITC, Pharmingen), anti-VEGFR(KDR)-FITC (Pharmingen), anti-CD45-FITC (Becton Dickinson Immunocytometry Systems, Mountain View, California, USA), as well as with lineage antibodies against anti-CD3, CD4, CD8, CD14, CD19, CD33, CD38 (all from Pharmingen). Isotype-matched mouse immunoglobulin served as a control. Samples were incubated at 4°C for 20 minutes, and cells were subsequently washed in PBS containing 1% heat-inactivated FCS and 0.1% sodium azide. Cells were analyzed using a FACS Calibur flow cytometer and Cell Quest software (Becton Dickinson). Each analysis included at least 5000–10000 events, and a light-scatter gate was defined to eliminate cell debris from the analysis. The percentage of CD133^+^ cells was assessed after correction for the percentage of cells reactive with the isotype control.

### GEArray gene expression and RT-PCR analysis of human CD133+ cells isolated from normal and DMD blood tissues

Total RNA was isolated from cells using the Trizol reagent (Invitrogen) as described by the manufacturer. Aliquots of total RNA (3 µg) were used for analysis of the human endothelial and muscular gene expression profile with GEArray technology (SuperArray Inc, Bethesda, MD) and RT-PCR analysis. The biotin deoxyuridine triphosphate (dUTP)-labelled cDNA probes were generated by the GEArray Ampolabeling-LPR kit. Random primers were incubated with total RNA at 70°C for 3 min for annealing, and samples were then reverse transcribed to cDNA at 37°C for 25 min. The cDNAs were amplified by PCR using gene-specific primers and biotin 16-UTP (Roche). PCR was performed according to the following protocol: 85°C for 5 min, 30 cycles of 85°C for 1 min, 50°C for 1 min, and 72°C for 1 min, and the final step at 72°C for 5 min. After prehybridation, membranes were incubated with biotin-labelled cDNAs overnight at 60°C; membranes were then washed and blocked with GEAblocking solution. The chemiluminescent detection was performed with alkaline phosphatase-conjugated streptavidin and CDP-star substrate, and visualized by exposure to electrochemilumiscence film. Data were acquired with a scanner (Epson), the digital image was converted to raw data file using the ScanAlyze Software, and the acquired data were analyzed with GEArray Analyzer Software (SuperArray). The expression level of each gene was compared to the levels of the housekeeping gene glyceraldehyde 3-phophate dehydrogenase (GAPDH) (3 spots in each array), whereas negative values were transformed to zero. Expression of human myogenic markers in CD133+ cells was also investigated by RT-PCR as previously described [16]. Only samples positive for GAPDH expression were considered, and we designed specific human myogenic primers in unique regions based on deposited human sequences. Total RNA was extracted from cells or muscles of mice using the TrizolReagent according to the manufacturer's protocol (Gibco BRL, Life Technologies). First strand cDNA was prepared using the Super Script First Strand Synthesis System for RT-PCR (Invitrogen, Life Technologies) starting from 2 µg total RNA with oligo(dT)_12-18_ priming. For direct amplification of human markers, primers were specifically designed for regions of human sequences that did not display homology with mouse mRNA sequences [16]. PCR was performed under the following conditions: 94° 5 min, 35 cycles at 94° for 40 sec, 68° for 40 sec, and 72° for 1 min.

### *In vitro* myo-endothelial potential of the human CD133+ subpopulations from normal and dystrophic blood tissues

CD133^+^ subpopulations isolated from normal and DMD blood tissues were plated at a density of 10^5^ cells/well in Falcon 6-well tissue culture plates in proliferation medium (PM) consisting of DMEM/F-12 (1∶1), 20% FBS, HEPES buffer (5 mM), glucose (0.6%), sodium bicarbonate (3 mM), and glutamine (2 mM). The following cytokines were added to the PM: stem cell factor (SCF, 100 ng/mL; TEBU, Frankfurt, Germany), vascular endothelial growth factor (VEGF, 50 ng/mL; TEBU) and leukemia inhibiting factor (LIF, 20 ng/ml; R&D Systems, Inc). Cells were passed every 8 days. To determine the myogenic potential, CD133 positive-derived cells were co-cultured with C2C12 murine myoblasts at a ratio of 5∶1 in differentiation medium (DM) consisting of Ham's F10 supplemented with 5% FBS, 10 ng/mL epidermal growth factor (EGF), 10 ng/mL platelet derived growth factor (PDGF-B) and antibiotics as previously described [16]. In these experiments, the sorted subpopulations of human CD133+ cells derived from the blood of normal and DMD subjects were labeled according to their expression of human lamin A/C. After 14 days of culture, the presence of chimeric human/murine myotubes was evaluated by immunostaining for desmin, slow myosin heavy chain (MyHCs) and human lamin A/C. The percentage of differentiated myotubes containing two or more nuclei expressing lamin A/C (i.e., fusion index of the human blood-derived CD133+ cells) was assessed. To determine whether CD133 positive subpopulations exhibited endothelial stem cell characteristics, sorted normal and DMD CD133+ cells were plated (∼10^3^ cells) in the presence of M199 (Gibco BRL) supplemented with 20% FBS (HyClone), vascular endothelial growth factor (10 ng/mL, Sigma), FGF (5 ng/mL, human recombinant basic FGF; Sigma), heparin (5 U/mL), penicillin (100 U/mL), streptomycin (100 µg/mL), and fungizone (0.25 µg/mL). Cells were placed on 12-well plates coated with 0.2% gelatin and incubated at 37°C in a humidified environment with 5% CO_2_. This process resulted in the attachment of mostly monocytes or mature endothelial colonies on the plates. After 4 to 5 days, non-adherent cells were transferred to other wells coated with 0.2% gelatin and grown in endothelial growth medium. Endothelial colonies with tubule formations were identified with primary mouse antibodies against human Ve-cadherin (1∶100; Becton Dickinson) and CD31 (1∶100; Chemicon) and visualized using an HRP-coupled secondary antibody (Bio-Rad Laboratories) in PBS containing 0.6 mg/ml diaminobezidine (Sigma Chemical Co). HUVECs were used as a positive control for Ve-cadherin staining. Quantitative analysis of total tubule formations expressing Ve-cadherin or CD31 antigens was performed with the AngioSys software (TCS CellWorks, Cat No. ZHA-1800).

### Clinical evaluation and follow-up of DMD patients

All DMD patients included in the study were diagnosed by clinical examination, muscle pathology, dystrophin analysis (western blot) and immunohistochemistry, and screened for mutations in the dystrophin gene. No patients were receiving steroid drugs at inclusion in this study. Sixty patients were evaluated; two were on ACE inhibitors and two were on carnitine. Muscle strength was estimated by manual muscle testing using the *Medical Research Council (MRC) Scale*. We tested the following muscle groups: neck flexors and extensors, shoulder abductors, elbow flexors and extensors, wrist flexors and extensors, trunk flexors and extensors, hip flexors, extensors, abductors, adductors, ankle flexors and extensors. As previously assessed [19], we then calculated the total muscle strength (% MRC) as % MRC = sum of grade score×100/number of muscles tested×5. The assessment of muscle strength of each subject was performed by two physiotherapists with expertise in neuromuscular disorders. Complete cardiac and respiratory evaluations were obtained from physical examination, chest X-ray, electrocardiography, M-mode, bidimensional and Doppler echocardiography and spirometry.

A clinical follow-up was performed in a subgroup of 19 patients by repeating the entire assessment 12 and 24 months later.

### Statistical analysis

Data were expressed as means±standard deviation (SD). Comparisons between DMD and controls were analyzed by 2 tailed Student's *t*-test (non-parametric) The univariate association between the levels of CD133+CXCR4+CD34- and CD133+CXCF4+CD34+ subpopulations with other variables was assessed using linear regression analysis. The dependent variable in this model was the continuous variable level of the subpopulation. Independent variables included patient age, muscle strength, the percentage of ejection fraction (EF%), and forced vital capacity (FVC) values. A probability of less than 5% was considered significant. These statistical analyses were performed using Prism Graphpad 4.0 (Graphpad, CA, USA) software.

To identify predictors of changes in colony counts of endothelial progenitor cells in a multivariate setting, we used multiple linear regression (SPSS) on specific variables.

## Results

### Quantitative studies of CD133+ stem cells and correlation with the DMD phenotype

**Figure 1:**
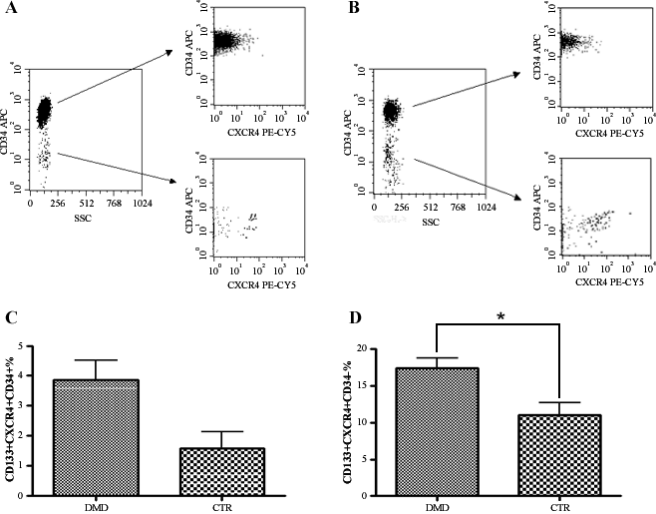
CD133+ cells were isolated from the peripheral blood of 70 DMD patients and 20 age-matched control subjects and analyzed by flow-cytometry. Representative panels show the CD133+CXCR4+CD34+ subpopulation in healthy subjects (mean percentage±SD, 1.58±2.39 of total CD133+ cells) (upper right panel in A) and in DMD patients (3.87±0.63)(lower right panel in B). A subpopulation of CD133+CXCR4+CD34-cells was significantly increased in DMD patients (lower right panel in B) compared with healthy controls (lower right panel in A) (mean percentage±SD, 17.38±1.38 vs. 11.0±1.70 of total CD133+ cells). (C) Histogram showing the percentages of CD133+CXCR4+CD34+ cells of healthy controls compared to DMD patients. (D) Histogram demonstrating the percentages of CD133+CXCR4+CD34- cells in healthy controls compared with to DMD patients. *Significance (*P* < 0.05).

**Figure 2:**
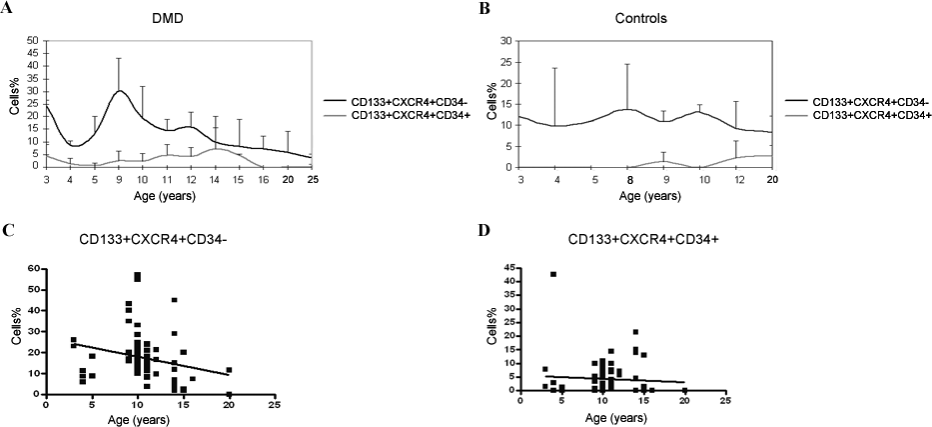
Levels of CD133+CXCD4+CD34- and CD133+CXCX4+CD34+ subpopulations are shown in DMD patients (A) and healthy controls (B) stratified for age. Levels of CD133+CXCR4+CD34- cells were constantly higher than CD133+CXCX4+CD34+ cells in both DMD patients and controls. DMD patients showed a nadir at the age of 4 years and one at the age of 9, with an overall tendency towards reduction with increasing age. Linear regression analysis of DMD patient data revealed a significant negative correlation (r^2^ = 0.056; P = 0.045) between the level of CD133+CXCR4+CD34- cells and age (C), while no significant correlation (r^2^ = 0.003; P = 0.63) between the level of CD133+CXCR4+CD34+ cells and age was observed (D).

**Figure 3:**
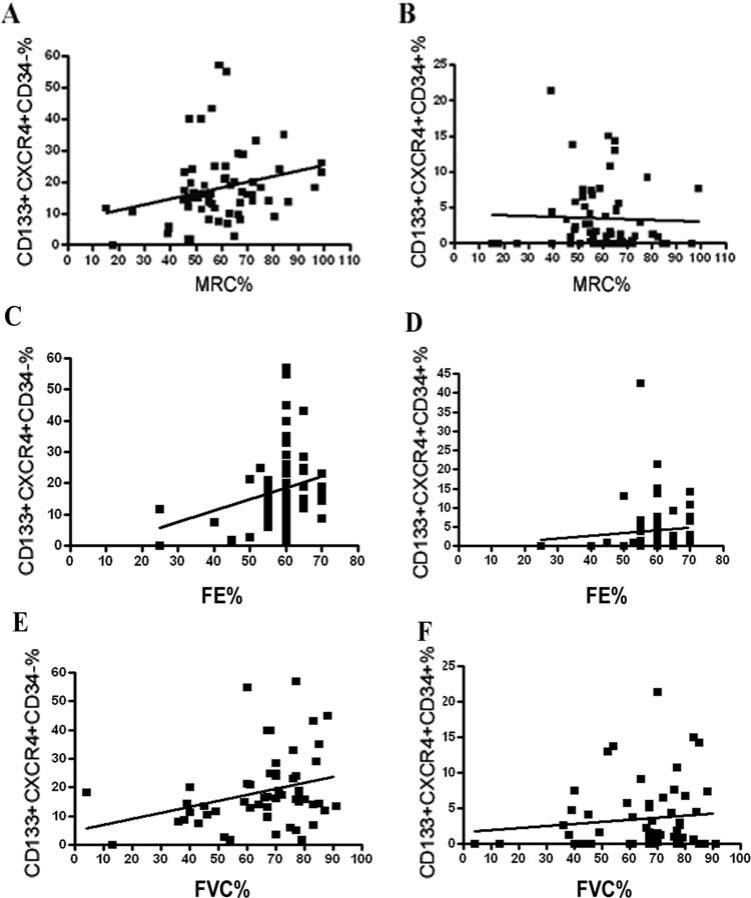
Correlation of CD133+CXCX4+CD34- and CD133+CXCX4+CD34+ subpopulations with MRC%, FE% and FVC% in DMD patients. A positive correlation was observed between the level of CD133+CXCX4+CD34- cells and MRC percentage (r^2^ = 0.065; P = 0.046)(A), FE% (r^2^ = 0.065; P = 0.043) (C), and FVC% (r^2^ = 0.089; P = 0.025) (E). No significant correlation was observed between the level of CD133+CXCX4+CD34+ cells and the above variables (B, D, and F).

CD133+ cells were isolated from a total of 70 patients with Duchenne muscular dystrophy recruited during routine clinical assessment at our Neuromuscular Centre; cells from 30 normal subjects were analyzed as controls. The mean (±SD) age of Duchenne patients was 10.66±3.81 (range 3 to 25). The frequency of mutations in the dystrophin gene in our DMD population was as follows: 35 patients (50%) had a mutation in the central region, 9 (12.86%) a deletion in the amino terminal region, 11 (15.71%) had other mutations (6 had deletions in the hot spot regions, 3 had a point mutation, and 2 had a duplication); fifteen patients (21.43%) had no identified mutations. We only analyzed the levels of CD133+ cells in DMD patients not taking steroid medication. Of the 70 patients, 12 had never initiated steroid therapy, while the remaining patients had stopped steroid therapy prior to assessment. CD133+ cells isolated from healthy subjects and DMD patients were characterized for the expression of several stem cell markers. Blood-derived CD133 positive cells represented less than 0.2% of the total mononucleated blood-derived cells in both normal and dystrophic patients (range 0.04–0.1% in normal samples and 0.06–0.2% in dystrophic samples). Hoechst 33342 staining of blood-derived CD133+ cells did not reveal the presence of the SP fraction within these cells (data not shown). More than 98% of the normal and dystrophic blood-derived CD133+ cells have a lineage negative phenotype (CD4-CD8-CD3-CD19-CD33-CD38-). Further analysis of a set of surface markers, such as CXCR4, CD34 and CD45, revealed these cells to be heterogeneous. In normal blood, 98% of CD133+ cells also expressed the CD34 antigen, whereas approximately 83–90% of dystrophic blood-derived CD133+ cells also expressed CD34. Moreover, approximately 30–45% of CD133+ cells isolated from normal and dystrophic blood tissues co-expressed CXCR4. Analysis of the percent of CD133+CXCR4+CD34+ subpopulation did not reveal any significant difference in DMD patients compared to healthy controls (3.87±0.63 in DMD subjects vs. 1.58±2.39 in controls; P = 0.12)(Fig. 1). Surprisingly, we observed that CD133+CXCR4+CD34- cells were significantly increased in DMD patients compared to healthy controls (17.38±1.38 vs. 11.0±1.70; P = 0.03)(Fig. 1). The percent of CD133+CXCR4+CD34-cells did not show any significant variance among patients with different mutations. We then analysed the levels of both subpopulations among the different age groups of the DMD patients and controls. Levels of CD133+CXCR4+CD34- cells were consistently higher than the levels of CD133+CXCR4+CD34- cells in both the DMD patients and controls, and tended to decline with advancing age in DMD subjects (Fig. 2A and B). We noticed that the levels of CD133+CXCR4+CD34- and CD133+CXCR4+CD34+ cells were quite stable over time in the controls, while in the DMD patients, the CD133+CXCR4+CD34- cells peaked at the age of 9 (Fig. 2A). Linear regression analysis showed a significant negative correlation between age and CD133+CXCR4+CD34- levels (r^2^ = 0,056; P = 0.045)(Fig. 2C). Linear regression analysis between the levels of circulating CD133+CXCR4+CD34- cells and MRC% values showed that higher levels of CD133+CXCR4+CD34- cells corresponded to higher muscle strength (r^2^ = 0.065; P = 0.046)(Fig. 3A). The same significant positive correlation was detected between percentage of cells and EF (r^2^ = 0.065; P = 0.043) and FVC% (r^2^ = 0.089; P = 0.025)(Fig. 3C and E, respectively).

Finally, multivariate regression analysis was performed to determine whether the number of CD133+CXCR4+CD34- cells was associated with age, MRC%, EF, or FVC%. This analysis demonstrated that EF was an independent predictor of the number of CD133+CXCR4+CD34- cells (P<0.001).

### Analysis of clinical severity groups and disease progression

**Figure 4:**
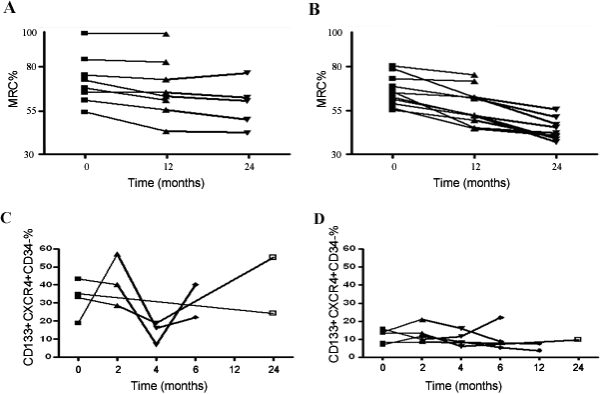
Representation of 2 different disease courses in 19 DMD patients stratified into 2 groups, in which a 24-month follow-up was available according to the results obtained from linear regression analysis between age and levels of the CD133+CXCR4+CD34- cells. MRC% scores are shown over time in 8 patients with a percentage of the cells localized above the threshold level for the corresponding age (A and C) and in 11 patients with values below the regression line during a total follow-up period of 24 months (B and D). At the end of the observation period, in the first group of DMD patients, the mean MRC% decreased by approximately 14%, and the total clinical score approximately 2.25 points. In the second group, the MRC% decreased by approximately 25%. In a subgroup including 4 DMD patients of the first group and in 5 DMD patients of the second group, we measured CD133+CXCR4+CD34- cells levels. Differences between the baseline values of CD133+CXCR4+CD34- cells and those at 2, 4, 6, 12 and 24 months are shown for individual patients in E and F. Despite the significant increase of CD133+CXCR4+CD34- cells compared to the patients of the second group (mean value±SD: 31.36%±14.67% vs. 11.12%±4.9%; P<0.0001), the 4 patients in group 1 displayed major intra-individual variability between successive measurements.

To assess the predictive power of CD133+CXCR4+CD34- cell levels, we investigated whether the levels of these cells correlated with the course of the disease. We stratified the DMD patients into two groups according to the results obtained from linear regression analysis of age and levels of CD133+CXCR4+CD34- cells, and then performed a detailed follow-up of 12 or 24 months on a subgroup of 19 DMD patients. Of these patients, 8 were categorized in the first group as exhibiting a percentage of CD133+CXCR4+CD34- cells localized above the threshold level for their corresponding age (mean age±SD: 9.17±3.01), while 11 patients were in the second ground, showing values below the regression line (mean age±SD: 10.91±3.04). Five patients in the first group and nine in the second completed the 24-month period of observation. In the first group, 3 patients were wheelchair bound at the beginning of the follow-up (patients were 8.8, 10.4, and 13.4 years old). Five patients were still ambulant (2.9, 8.1, 9.4, 10.2 and 10.3 years), and of these, 2 lost ambulation during the observation period at 10.5 and 10.9 years of age. Thus, the mean age at loss of ambulation in this group was 10.04±1.89. A worsening of muscle strength was observed during the observation period: the mean MRC% was 72.54±14.02 at the onset (n = 8), 67.87±16.97 after 12 months (n = 8), and 58.53±12.99 after 24 months (n = 5)(Fig. 4A). At the beginning of the follow up, only one patient from the first group showed cardiac ECG abnormalities, while echocardiographic findings were normal in all patients. Spirometry measures indicative of a mild restrictive lung disease were detected in 2 patients. During the follow-up, we observed a preservation of cardiac and respiratory functions. In particular, after 12 months, the spirometric analysis showed the appearance of a mild restrictive pattern only in two patients. After the next 12 months, no further deteriorations were observed. The ECG findings showed sinus tachycardia in one patient and conduction abnormalities in another patient. Echocardiographic data remained normal throughout the follow up. In the second group, only one patient was still ambulant (4.8 years of age), and the other patients had already lost the ability to walk independently. The mean age at loss of ambulation was 10.21±1.9. At the beginning of the study, patients in group two had a mean age of 11.52±3.4 (ranging from 9.5 to 14.8 years). The mean MRC% changed from 66.09±8.5 (n = 11), to 57.42±1.33 (n = 11), to 40.97±6.22 (n = 9)(Fig. 4B). At the beginning of the observation period, 8 patients did not show cardiac abnormalities by ECG and echocardiography assessments. Of these patients, 3 developed ECG abnormalities, such as sinus tachycardia and conduction abnormalities. Echocardiographic findings remained normal until the end of the study (24 months for 2 patients and 12 months for the third). Two patients had EGC abnormalities from the beginning of the study: a complete right bundle branch block and conduction abnormalities with no abnormalities at echocardiography in both cases until the end of the study. One patient showed left ventricular regional wall motion abnormalities and mitral valve prolaps from the outset. He then presented a rapid deterioration of systolic function with an EF of 35% and treatment with ACE inhibitors was initiated. Regarding respiratory function, 6 patients presented baseline normal spirometric characteristics. In 4 of these patients, the FVC remained within normal range values until the last evaluation, while the other two showed a progressive worsening and fall of the FVC that reached 45% predicted in one patient and 50% predicted in the second after 24 months. In the other 5 patients, at the outset of the study, a mild reduction in spirometric variables and restrictive impairment was seen, which increased until the end of the study (FVC range between 47% and 55%).

In a subgroup of 10 patients (n = 4 in the first group and n = 6 in the second group), we repeated the analysis of the levels in the two subpopulations at different times. The changes in CD133+CXCR4+CD34- percentages for individual patients are shown in Figure 4 (C and D). Throughout the study, the levels of CD133+CXCR4+CD34- cells were significantly higher in the group of DMD patients with a slow progressive disease course (mean value±SD: 31.36%±14.67% vs. 11.12%±4.9%; P<0.0001). Otherwise, the levels varied in a range of 7–55% in the first group of patients with a mild course of disease, and between 3.6–21.95% in the second group of patients with severe disease progression. We also observed great intra-individual variability in two subjects of the first group in particular. These data suggest that serial measurements of the CD133+CXCR4+CD34- cells (not less than three) should be performed at standardized times for a greater number of subjects.

### Myo-endothelial potential and migrating phenotype of blood-derived CD133 subpopulations isolated from healthy controls and DMD patients

To determine whether the differences in the levels of circulating CD133+CXCR4+CD34- stem cells observed in mild and severe DMD patients correlate to a different myo-endothelial potential of these cells, myogenic and angiogenic assays were performed. The CD133+CXCR4+CD34- cells were sorted with a dual-laser FACS Vantage SE (Becton Dickinson Immunocytometry Systems) and the purity of cell sorting was confirmed as 98% in all experiments. Using a human Stem Cell Gene Array, we found that the CD133+CXCR4+CD34- sorted cells expressed genes reflective of vascular progenitors, including *CD31, FZD 2/3/4/7, CXCR4, LIFR,* and *Notch* promoter 1 (data not shown). To verify the endothelial potential of sorted cells, the CD133+CXCR4+CD34- cells from normal and dystrophic blood were cultured in the presence of endothelial medium (*End*) as indicated in Material and Methods. Under these conditions, we observed the formation of colonies with spindle-like cells expressing Ve-cadherin and CD31 endothelial markers from both normal and dystrophic CD133+CXCR4+CD34- cell populations. We also observed that in *End* culture, no colonies formed when fewer than 300 cells/well were seeded, indicating the requirement of cell–cell interactions in these conditions. Since circulating CD133+ stem cells did not differentiate spontaneously into myotubes in vitro [16], we investigated the expression of human myogenic markers in normal and dystrophic sorted CD133+CXCR4+CD34- cells co-cultured with C2C12 murine myoblasts. RT-PCR analysis revealed the expression of *M-cadherin*, *Myf-5*, *Myogenin*, *Pax7*, *MyoD* and *MRF4* in normal and DMD CD133+CXCR4+CD34- cells after 7 days of co-culture. The myogenic rate of the CD133+CXCR4+CD34- stem cells was similar in DMD patients (4.13%±1.25% of the total human lamins A/C positive cells per well; n = 10) compared to healthy controls (3.82%±0.9% of the total human lamins A/C positive cells per well; n = 10), whereas the angiogenic rate of these cells was selectively increased with no significant differences in mild DMD patients (32.47%±11.7% of the total human lamins A/C positive cells per well; n = 10) versus healthy control subjects (27.0%±3.71% of the total human lamins A/C positive cells per well; n = 10) and versus severe DMD patients (27.57%±6.14% of the total human lamins A/C positive cells per well; n = 10). According to our previous observations [20], blood-derived CD133+ stem cells express several adhesion molecules involved in cell migration through vessels into the skeletal muscle tissue. To assess whether the circulating CD133+CXCR4+CD34- cells isolated from normal and DMD patients were in a migratory state, we analyzed them for the expression of molecules that are expressed on the surface of stem cells with high migratory capacity, as previously described [20]. The number of CD133+CXCR4+CD34- cells with surface expression of L-selectin or vascular cell adhesion molecule 1 (VCAM-1) was markedly increased in DMD patients compared with healthy controls (*P*<0.01 and *P*<0.001, respectively)(data not shown). Moreover, the level of circulating CD133+CXCR4+CD34- stem cells positive for these migratory markers was higher in patients with mild phenotype than in patients with a severe condition, and this showed a significant correlation with the clinical score (r = 0.241, *P*<0.05)(data not shown). Negative linear regression between the levels of circulating CD133+CXCR4+CD34- stem cells expressing L-selectin and VCAM-1 and the age of DMD patients was detected (r = −0.145, *P*<0.05 and r = −0.217, *P*<0.05, respectively)(data not shown).

## Discussion

Recent works from several laboratories have identified various serum markers and cellular markers in predicting mortality and morbidity in a wide variety of diseases, such as cardiovascular disease and tumor pathologies [13]–[15], [21]–[23]. In the field of muscular dystrophies, no indicators have been identified thus far, hence the importance of a finding a marker that may help identify patients at increased risk for faster disease progression.

The main aim of our study was to determine whether the level of circulating CD133+ stem cells was related to the disease status of DMD. This finding would have a significant impact on patients' treatment and management.

We selected a DMD population composed of subjects not undergoing steroid therapy to avoid potential confusing variables. Moreover, steroids are well known to influence the development, differentiation and homeostasis of a large number of cells. In particular, glucocorticoids (GC) affect the differentiation of several types of stem cells such as mesenchymal [24] and neural stem [25] cells *in vitro,* and also have an effect on the proliferation and maturation of human peripheral blood stem cells *in vitro* and *in vivo* [26]. The migratory capacity and relative baseline levels of CD133+ cells may be influenced by chronic steroid therapy. In fact, as we recently demonstrated [20], CD133+ stem cells express several adhesion molecules, such as CD44, LFA-1, PSGL-1, α4-integrins, L-selectin, and chemokine receptor CCR7, that provide the physical connection with the activated endothelium in the DMD inflamed muscle. Steroids could affect the interaction between CD133+ cells and the endothelium due to their anti-inflammatory action on the DMD muscle or cause a direct change in the expression of surface adhesion molecules on CD133+ cells.

Analysis of the number of circulating CD133+ cells from the peripheral blood of DMD patients revealed an interesting course of the CD133+CXCR4+CD34- subpopulation. We found that the percentage of CD133+CXCR4+CD34- cells was increased in DMD patients compared with healthy controls. The mean levels of CD133+CXCR4+CD34- in DMD subjects showed a tendency to decrease with advancing age, although there were two nadirs between the ages of 4–5 years and 11–12 years. Both of these ages correspond to a crisis in Duchenne muscles resulting in the onset of symptoms in the first time period and the loss of independent ambulation in the second period. Our hypothesis is that the circulating levels of CD133+ cells at these particular stages are lower as a result of a major homing in the injured muscle.

The clinical severity of each patient was assessed by manual muscle testing and respiratory and cardiac function measurements. From the literature, particular consideration has been given to monitoring muscle strength and the functional abilities of DMD patients, and several standardized scales have been developed [19], [27]. However, in order to determine the clinical status and the extent of the disease progression, it is also important to assess, as objectively as possible, cardiac [28]–[30] and respiratory function. Pulmonary function assessment, such as forced vital capacity (FVC), is an acknowledged clinically relevant endpoint and the rate of decline in FVC was found to be useful in predicting the life expectancy of DMD patients [7], [8]. Linear regression analysis showed a positive correlation between the levels of circulating CD133+CXCR4+CD34- cells and muscle strength, EF% and FVC% in DMD patients. A strong correlation was also confirmed between the levels of circulating CD133+CXCR4+CD34- cells and the rate of disease progression. In fact, the levels of CD133+CXCR4+CD34- cells were greater in DMD patients with a slow progressive course compared to DMD patients who showed a rapid disease course. Multivariate analysis between the number of CD133+CXCR4+CD34- cells and MRC%, FE% and FVC% confirmed that all these variables are predictors of the number of CD133+CXCR4+CD34- cells, with FE% as the best predictor. These data suggest that the levels of circulating CD133+CXCR4+CD34- cells could be a biomarker for DMD.

Moreover, we observed that the nature of the dystrophin gene mutation did not affect the levels of both CD133+CXCR4+CD34- and CD133+CXCR4+CD34+ cells in the DMD subjects analyzed in this study. In fact, comparison of patients based on the genetic mutation did not reveal any significant difference with respect to the percentage of these two subpopulations.

The nature and size of our study does not permit us to determine whether low levels of CD133+CXCR4+CD34- cells can accurately predict the progression of disease. On the other hand, for disorders in which the patient population is small, it is critical to obtain reliable data working with a small sample size. Further research will need to focus on determining the precise clinical relevance of the level of CD133+CXCR4+CD34- cells, and if and how these correlate with quality of life, time of death, and other life-changing events (e.g., using a wheelchair). In this study, we also investigated the myogenic and endothelial potential of CD133+CXCR4+CD34- cells isolated from the peripheral blood of healthy and DMD subjects. Freshly isolated CD133+CXCR4+CD34- cells from normal and dystrophic blood express several myogenic and endothelial markers, suggesting similar myo/endothelial potential. Moreover, the CD133+CXCR4+CD34- cells derived from the DMD blood cultured in the presence of endothelial medium give rise to a higher number of endothelial colonies than those from the normal counterpart, indicating that these cells probably receive more specific signals for endothelial differentiation from DMD tissues. There are several possibilities that might account for the increase in angiogenic capacities of this subpopulation of CD133+ stem cells. First, it has been established that endothelial progenitors can be mobilized from the bone marrow to blood in response to a variety of inflammatory cytokines like those released in dystrophic skeletal muscle tissues, which could explain the increased levels of CD133+CXCR4+CD34- stem cells in DMD patients. Indeed, previous studies have suggested that activated endothelial progenitors express several adhesion molecules, such as E-selectin and VCAM-1 [31], involved in the migration from the blood. Moreover, we previously demonstrated that VCAM-1 blockade prevents blood-derived CD133+ accumulation in dystrophic muscle and that the VCAM-1-VLA-4 adhesion receptor pair has a critical role in the recruitment of these stem cells to dystrophic muscle vessels [16]. For these reasons, we stratified the subpopulations of circulating CD133+ stem cells based on the expression of several adhesion molecules. We found that the number of CD133+CXCR4+CD34- cells with surface expression of L-selectin and VCAM-1 was markedly increased in DMD patients compared with healthy controls and with a preponderance of these cells in patients with a mild phenotype. Moreover, the levels of CD133+CXCR4+CD34- cells expressing L-selectin and VCAM-1 decrease with age and course of the disease. We believe all these data suggest that the circulating CD133+CXCR4+CD34- stem cells have a principal role in the vascular homeostasis of the dystrophic skeletal muscle and may partially contribute to muscle regeneration. We further speculate that continuous muscle degeneration of dystrophic muscles may lead to progressive injury of muscle fibers and vessels with consequent depletion of these myo/endothelial circulating progenitor cells. Future studies will therefore be needed to determine whether this postulated muscle-vessel injury–induced exhaustion of circulating myo/endothelial progenitor cells is a factor in the pathogenesis of Duchenne muscular dystrophy disease.

## References

[1] Infante JP, Huszagh VA (1999). Mechanisms of resistance to pathogenesis in muscular dystrophies.. Mol Cell Biochem.

[2] Hoffman EP, Brown RH, Kunkel LM (1987). Dystrophin: the protein product of the Duchenne muscular dystrophy locus.. Cell.

[3] Hoffman EP, Morgan JE, Watkins SC, Partidge TA (1990). Somatic reversion/suppression of the mouse mdx phenotype in vivo.. J Neurol Sci.

[4] Baumbach LL, Chamberlain JS, Ward PA, Farwell NJ, Caskey CT (1989). Molecular and clinical correlations of deletions leading to Duchenne and Becker muscular dystrophies.. Neurology.

[5] Muntoni F, Torelli S, Ferlini A (2003). Dystrophin and mutations: one gene, several proteins, multiple phenotypes.. Lancet Neurol.

[6] Finsterer J, Stollberger C (2003). The heart in human dystrophinopathies.. Cardiology.

[7] Eagle M, Baudouin SV, Chandler C, Giddings DR, Bullock R (2002). Survival in Duchenne muscular dystrophy: improvements in life expectancy since 1967 and the impact of home nocturnal ventilation.. Neuromuscul Disord.

[8] Phillips MF, Quinlivan RC, Edwards RH, Calverley PM (2001). Changes in spirometry over time as a prognostic marker in patients with Duchenne muscular dystrophy.. Am J Respir Crit Care Med.

[9] Lorenz MW, von Kegler S, Markus HS, Sitzer M (2006). Carotid intima-media thickening indicates a higher vascular risk across a wide age range: prospective data from the Carotid Atherosclerosis Progression Study (CAPS).. Stroke.

[10] Wilson P, D'Agostino R, Levy D, Belanger A, Silbershatz H (1998). Prediction of coronary heart disease using risk factor categories.. Circulation.

[11] Makita S, Nakamura M, Hiramori K (2005). The association of c-reactive protein levels with carotid intima-media complex thickness and plaque formation in the general population.. Stroke.

[12] De Groot E, Hovingh GK, Wiegman A, Duriez P, Smit AJ (2004). Measurement of arterial wall thickness as a surrogate marker for atherosclerosis.. Circulation.

[13] Boos CJ, Lip GY, Blann AD (2006). Circulating endothelial cells in cardiovascular disease.. J Am Coll Cardiol.

[14] Werner N, Kosiol S, Schiegl T, Ahlers P, Walenta K (2005). Circulating endothelial progenitor cells and cardiovascular outcomes.. N Engl J Med.

[15] Fadini GP, Schiavon M, Cantini M, Baesso I, Facco M (2006). Circulating progenitor cells are reduced in patients with severe lung disease.. Stem Cells.

[16] Torrente Y, Belicchi M, Sampaolesi M, Pisati F, Meregalli M (2004). Human circulating AC133(+) stem cells restore dystrophin expression and ameliorate function in dystrophic skeletal muscle.. J Clin Invest.

[17] Miraglia S, Godfrey W, Yin AH, Atkins K, Warnke R (1997). A novel five-transmembrane hematopoietic stem cell antigen: isolation, characterization, and molecular cloning.. Blood.

[18] Gallacher L, Murdoch B, Wu DM, Karanu FN, Keeney M (2000). Isolation and characterization of human CD34(−)Lin(−) and CD34(+)Lin(−) hematopoietic stem cells using cell surface markers AC133 and CD7.. Blood.

[19] Scott E, Mawson SJ (2006). Measurement in Duchenne muscular dystrophy: considerations in the development of a neuromuscular assessment tool.. Dev Med Child Neurol.

[20] Gavina M, Belicchi M, Rossi B, Ottoboni L, Colombo F (2006). VCAM-1 expression on dystrophic muscle vessels has a critical role in the recruitment of human blood-derived CD133+ stem cells after intra-arterial transplantation.. Blood.

[21] Guven H, Shepherd RM, Bach RG, Capoccia BJ, Link DC (2006). The number of endothelial progenitor cell colonies in the blood is increased in patients with angiographically significant coronary artery disease.. J Am Coll Cardiol.

[22] Duda DG, Cohen KS, di Tomaso E, Au P, Klein RJ (2006). Differential CD146 expression on circulating versus tissue endothelial cells in rectal cancer patients: implications for circulating endothelial and progenitor cells as biomarker for antiangiogenic therapy.. J Clin Oncol.

[23] Dome B, Timar J, Meszaros L, Raso E, Paku S (2006). Identification and clinical significance of circulating endothelial progenitor cells in human non-small cell lung cancer.. Cancer Res.

[24] Derfoul A, Perkins GL, Hall DJ, Tuan RS (2006). Glucocorticoids promote chondrogenic differentiation of adult human mesenchymal stem cells by enhancing expression of cartilage extracellular matrix genes.. Stem Cells.

[25] Sabolek M, Herborg A, Schwarz J, Storch A (2006). Dexamethasone blocks astroglial differentiation from neural precursors cells.. Neuroreport.

[26] Grafte-Faure S, Leveque C, Vasse M, Soria C, Norris V (1999). Effects of glucocorticoids and mineralcorticoids on proliferation and maturation of human peripheral bllod stem cells.. Am J Hematol.

[27] Scott OM, Hyde SA, Goddard C, Dubowitz V (1982). Quantitation of muscle function in children: a prospective study in Duchenne muscular dystrophy.. Muscle & Nerve.

[28] Brooke MH, Fenichel GM, Griggs RC, Mendell JR, Moxley R (1989). Duchenne muscular dystrophy: patterns of clinical progression and effect of supportive therapy.. Neurology.

[29] Angelini C, Fanin M, Freda MP, Martinello F, Miorin M (1996). Prognostic factors in mild dystophinopathies.. J Neurol Sci.

[30] Hyde SA, Steffensen BF, Floytrup I, Glent S, Kroksmark AK (2001). Longitudinal data analysis: an application to construction of a natural history profile of Duchenne muscular dystrophy.. Neuromuscul Disord.

[31] Del Papa N, Colombo G, Fracchiolla N, Moronetti LM, Ingegnoli F (2004). Circulating endothelial cells as a marker of ongoing vascular disease in systemic sclerosis.. Arthritis Rheum.

